# Temperature-modulated interactions between thermoresponsive strong cationic copolymer-brush-grafted silica beads and biomolecules

**DOI:** 10.1016/j.heliyon.2024.e34668

**Published:** 2024-07-20

**Authors:** Kenichi Nagase, Sayaka Suzuki, Hideko Kanazawa

**Affiliations:** a Graduate School of Biomedical and Health Sciences, Hiroshima University, 1-2-3 Kasumi, Minami-ku, Hiroshima, 734-8553, Japan; b Faculty of Pharmacy, Keio University, 1-5-30 Shibakoen, Minato, Tokyo, 105-8512, Japan

**Keywords:** Polymer brush, Thermoresponsive polymer, Chromatography, Hydrophobic interaction, Electrostatic interaction

## Abstract

Thermoresponsive polymer brushes have attracted considerable research attention owing to their unique properties. Herein, we developed silica beads grafted with poly(*N*-isopropylacrylamide (NIPAAm)-*co*-3-acrylamidopropyl trimethylammonium chloride (APTAC)-*co*-*tert*-butyl acrylamide (tBAAm) and P(NIPAAm-*co*-APTAC-*co*-*n*-butyl methacrylate(nBMA)) brushes. The carbon, hydrogen, and nitrogen elemental analysis of the copolymer-grated silica beads revealed the presence of a large amount of the grafted copolymer on the silica beads. The electrostatic and hydrophobic interactions between biomolecules and prepared copolymer brushes were analyzed by observing their elution behaviors via high-performance liquid chromatography using the copolymer-brush-modified beads as the stationary phase. Adenosine nucleotides were retained in the bead-packed columns, which was attributed to the electrostatic interaction between the copolymers and adenosine nucleotides. Insulin was adsorbed on the copolymer brushes at high temperatures, which was attributed to its electrostatic and hydrophobic interactions with the copolymer. Similar adsorption behavior was observed in case of albumin. Further, at a low concentration of the phosphate buffer solution, albumin was adsorbed onto the copolymer brushes even at relatively low temperatures owing to its enhanced electrostatic interaction with the copolymer. These results indicated that the developed thermoresponsive strong cationic copolymer brushes can interact with peptides and proteins through a combination of electrostatic and temperature-modulated hydrophobic interactions. Thus, the developed copolymer brushes exhibits substantial potential for application in chromatographic matrices for the analysis and purification of peptides and proteins.

## Highlights

• Thermoresponsive polymer brushes with strong cationic groups were developed.
• Two hydrophobic monomers were introduced on the copolymer brush.
• Insulin and albumin adsorbed on the copolymer brushes at high temperatures.
• Polymer brush interfaces can be used for separating peptides and proteins.

## Graphical abstract

**Figure:**
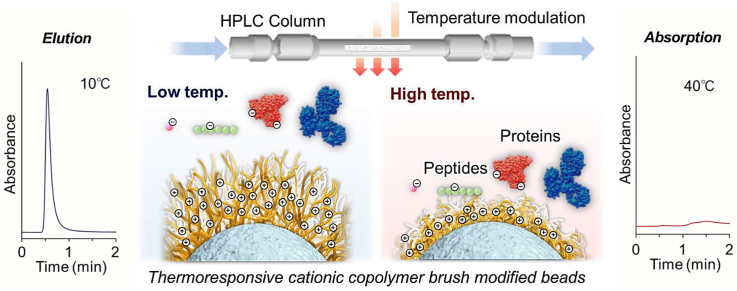

## 1 Introduction

Stimuli-responsive functional polymers have been investigated in the fields of polymer chemistry and materials chemistry [[1], [2], [3], [4], [5], [6]]. In particular, poly(*N*-isopropylacrylamide) (PNIPAAm) has garnered attention as a stimuli-responsive functional polymer. Poly(*N*-isopropylacrylamide) (PNIPAAm) exhibits temperature-dependent hydrophilic/hydrophobic behavior at low and high temperatures, attributed to hydration and dehydration, respectively [[7], [8], [9], [10], [11], [12], [13]]. This thermoresponsive property leads to PNIPAAm being exploited in various applications, such as thermoresponsive drug delivery [[14], [15], [16], [17], [18], [19], [20], [21], [22], [23], [24], [25], [26]], bioanalysis and biosensors [[27], [28], [29], [30], [31], [32], [33], [34], [35], [36], [37], [38]], nanoactutors [[39], [40], [41], [42]], bioseparation tools [[43], [44], [45], [46], [47], [48], [49], [50], [51], [52], [53], [54], [55]], cell separation materials [[56], [57], [58], [59], [60], [61], [62], [63], [64], [65], [66], [67], [68], [69], [70], [71]], and cell-culture substrates for tissue engineering [[72], [73], [74], [75], [76], [77], [78], [79], [80], [81], [82], [83], [84], [85], [86], [87], [88], [89], [90], [91]].

Based on the aforementioned advantages of PNIPAAm, the structures of PNIPAAm-modified interfaces have been investigated to achieve improved performance for the abovementioned applications. Various structures of grafted PNIPAAm fabricated using several grafting methods have been investigated. In particular, PNIPAAm brushes prepared via atom transfer radical polymerization (ATRP) have been attracting attention owing to their unique characteristics [[92], [93], [94], [95], [96], [97], [98]]. ATRP is one of the effective living radical polymerization methods that can control the polymer length with precision [99]. Polymer grafting through ATRP can facilitate the formation of a dense polymer brush layer on the substrate [100]. Thus, various polymer brushes have been developed via ATRP using various types of polymers [[101], [102], [103], [104], [105], [106]]. PNIPAAm brushes have been investigated because of their intrinsic properties, such as wettability and shrinkage, which are different from those of the PNIPAAm-modified substrates prepared using the conventional “grafting to” and “grafting from” radical polymerization methods [[107], [108], [109], [110], [111]].

One of the promising applications of PNIPAAm brushes is in temperature-responsive chromatography [44]. In this application, PNIPAAm-grafted beads are employed as the stationary phase. The hydrophobic interaction of PNIPAAm with the analyte in the chromatography column is modulated by varying the temperature, which induces the dehydration of PNIPAAm on the beads. The PNIPAAm-brush-grafted beads prepared via ATRP exhibit longer analyte retention times than the conventional PNIPAAm-grafted beads prepared using “grafting to” and “grafting from” methods. This is because the amount of the grafted PNIPAAm on the beads prepared via ATRP is considerably higher than that on the PNIPAAm beads prepared using conventional polymerization methods, which enhances the hydrophobic interaction of PNIPAAm with analytes.

The hydrophobicity of the polymer brush can be increased by incorporating a hydrophobic monomer, such as *n*-butyl methacrylate (nBMA) or *tert*-butylacrylamide (tBAAm), into the PNIPAAm structure. The hydrophobized-copolymer-brush-modified stationary phase can retain analytes via strong hydrophobic interactions compared with the results achieved with the PNIPAAm homopolymer brush [112].

In addition, thermoresponsive, ionic copolymer brushes formed via the copolymerization of an ionic monomer with PNIPAAm have been investigated [53,113]. Such thermoresponsive, ionic copolymer brushes are used in ion-exchange chromatography matrices for separating ionic analytes. The electrostatic interaction of the copolymer with analytes can be modulated by varying the temperature. This is because the ionization of the ionic group in the copolymer is affected by the hydrophobicity of the copolymer, which can be modulated by varying the temperature.

Furthermore, thermoresponsive protein-adsorption materials have been developed by incorporating ionic and hydrophobic monomers into PNIPAAm [114]. The electrostatic and hydrophobic interactions occur between the copolymers and proteins, resulting in temperature-modulated protein adsorption on the copolymers. In a previous study, thermoresponsive, strong, anionic copolymer-modified beads were developed using 2-acrylamido-2-methylpropanesulfonic acid (AMPS) as a strong acidic monomer as well as nBMA and tBAAm as hydrophobic monomers [48]. The adsorption capacity of the copolymer-brush-modified silica beads toward the antibody drug rituximab could be improved by varying the temperature. The protein-adsorption behavior was influenced by the ionic and hydrophobic monomers. Thus, selecting a proper hydrophobic monomer is crucial for improving the protein-adsorption property of modified silica beads.

In this study, we developed silica beads grafted with thermoresponsive, strong cationic copolymer brushes. The developed cationic copolymer brushes were employed for the separation analysis of acidic biomolecules, such as adenosine nucleotide, insulin, and albumin. The compound 3-acrylamidopropyl trimethylammonium chloride (APTAC) was used as the cationic monomer because it has a quaternary amine group and strong cationic property. In addition, two types of hydrophobic monomers, namely tBAAm and nBMA, were used to investigate the effect of hydrophobic monomers on the protein-adsorption property of the beads. The prepared copolymer brushes were characterized by observing the elution behavior of the acidic biomolecules.

## 2 Methods

### 2.1 Copolymer-brush preparation on silica beads

**Fig. 1:**
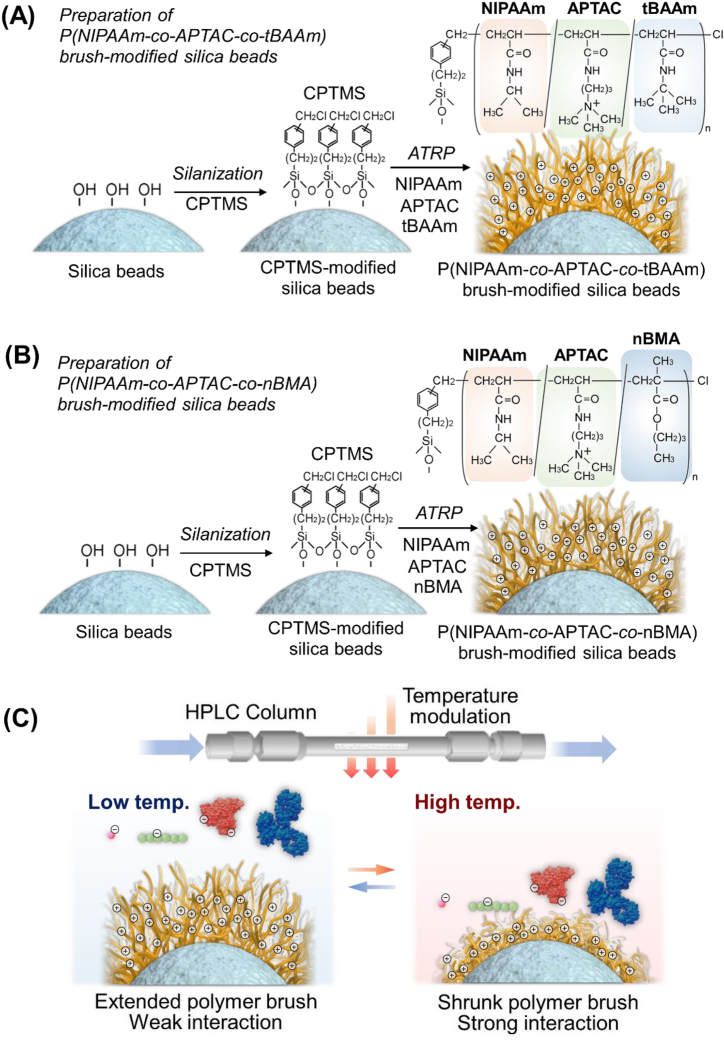
Schematic illustration of the thermoresponsive, strong, cationic copolymer brushes with two types of hydrophobic monomers and their interactions with peptides and proteins. (A) Scheme for the preparation of the P(NIPAAm-*co*-APTAC-*co*-tBAAm) brush on silica beads. (B) Scheme for the preparation of the P(NIPAAm-*co*-APTAC-*co*-nBMA) brush on silica beads. (C) Temperature-modulated interactions between the prepared polymer brush and small acidic molecules, peptides, and proteins in a high-performance liquid chromatography column.

All the reagents used in this study are described in Supplementary Materials. Thermoresponsive strong cationic copolymer brushes were prepared via ATRP on silica beads using various hydrophobic monomers (Fig. 1A and B). The silica beads (31.4 g) were activated using hydrochloric acid (250 mL) at 90 °C for 2 h, after which they were dried at 150 °C for 16 h. Subsequently, the beads were incubated at a relative humidity of 60 % by supplying humidified nitrogen gas. ((Chloromethyl)phenylethyl) trimethoxysilane (CPTMS; 8.25 mL; 33.3 mmol) was added to 613 mL of toluene. Thereafter, the solution was reacted with the activated silica beads, followed by silanization at 25 °C for 16 h under continuous stirring. Subsequently, the product was rinsed with toluene and dried at 110 °C for 3 h.

P(NIPAAm-*co*-APTAC-*co*-tBAAm) was grafted on the CPTMS-modified silica beads via ATRP (Fig. 1A). APTAC containing 25 wt% water (5.93 g, 21.5 mmol) was added to 440 mL of 2-propanol, and the polymerization inhibitor in the APTAC solution was removed using an inhibitor-remover column. NIPAAm (25.6 g; 227 mmol) and tBAAm (7.66 g; 60.2 mmol) were dissolved in the APTAC solution (299.6 mL) in a molar ratio of NIPAAm:APTAC:tBAAm = 75:5:20, and the total monomer concentration was 1.00 mol/L. The monomer solution was bubbled under Ar gas flow for 60 min to remove oxygen. Thereafter, tris 2-(dimethylamino)ethyl amine (Me_6_TREN) (1.55 g; 6.71 mmol), CuCl (593 mg; 5.99 mmol), and CuCl_2_ (80.5 mg; 0.599 mmol) were added. The CPTMS-modified beads (7.00 g) were added to the ATRP reaction solution, and ATRP was conducted at 25 °C for 16 h under continuous stirring. Subsequently, the beads were rinsed with acetone, the mixed solvent comprising methanol and a 50-mM ethylenediamine-*N,N,N′,N′*-tetraacetic acid solution (v:v = 1:1), and pure water, after which they were dried at 50 °C for 2 h. The prepared beads were denoted as NI-QA5-tB20 (NI represents NIPAAm, QA represents the quaternary amine of APTAC, and tB represents tBAAm; numbers 5 and 20 indicate the molar ratios of the monomers, respectively).

The P(NIPAAm-*co*-APTAC-*co*-nBMA) brush was prepared on the CPTMS-modified silica beads via ATRP (Fig. 1B). The polymerization procedure used for preparing the P(NIPAAm-*co*-APTAC-*co*-tBAAm) brush was employed again, except that nBMA replaced tBAAm as the hydrophobic monomer and the molar ratio was NIPAAm:APTAC:nBMA = 85:5:10. The prepared beads were denoted as NI-QA5-nB10, where nB represents nBMA.

The amounts of the modified CPTMS and the copolymer were measured via carbon, hydrogen, nitrogen (CHN) elemental analysis. The amount of the modified CPTMS on the silica beads was calculated using the following formula (1):

(1) $$\text{CPTMS}\;\text{amount}=\frac{\%C_{C}}{\%C_{C}\left(calcd\right)\times \left(1-\%C_{C}/\%C_{C}\left(calcd\right)\right)\times S}\text{,}$$

where %*C*_*C*_ represents the increase in the carbon composition through the silanization reaction, %*C*_*C*_ *(calcd)* represents the theoretical carbon content of CPTMS, and *S* represents the surface area of the unmodified silica beads (100 m^2^/g). The amount of the grafted copolymer was calculated using the following equation (2):

(2) $$\text{Copolymer}\;\text{brush}\;\text{amount}=\frac{\%C_{P}}{\%C_{P}\left(calcd\right)\times \left(1-\%C_{P}/\%C_{P}\left(calcd\right)-\%C_{C}/\%C_{C}\left(calcd\right)\right)\times S}\text{,}$$

where %*C*_*P*_ represents the increase in the carbon composition through ATRP and %*C*_*P*_ *(calcd)* represents the theoretical carbon content of the copolymer.

### 2.2 Temperature-modulated interactions between the copolymer brushes and biomolecules

The interaction of the polymer brush with biomolecules was analyzed using high-performance liquid chromatography (HPLC). The prepared copolymer-grafted silica beads were packed into a stainless steel column (inner diameter: 4.6 mm and column length: 50 mm). The prepared silica beads (0.8 g) were suspended in 2 mL of a mixed solvent comprising methanol and water (v:v = 1:1). Subsequently, the bead suspension was poured into the reservoir of the column packer that connected the stainless steel column. The methanol–water mixed solvent was allowed to flow into the column packer using an HPLC pump. The bead-packed column was washed using pure water at 40 °C for 18 h.

Adenosine nucleotides were employed as the small biomolecules, and their interactions with the copolymer brushes were investigated. The properties of the adenosine nucleotides are summarized in Table S1. Adenosine monophosphate (AMP; 0.2 mg), adenosine diphosphate (ADP; 1.2 mg), and adenosine triphosphate (ATP, 13.1 mg) were added to 4 mL of a 66.7-mmol/L phosphate buffer solution (pH: 7.0) in a glass vessel. The sample was prepared by filtering the solution through a syringe filter with a 0.45-μm pore diameter. The prepared column was equilibrated using a 66.7-mmol/L phosphate buffer solution (pH: 7.0) at 1.0 mL/min for 3 h. The elution behaviors of the adenosine nucleotides were observed at 254 nm at various temperatures using an HPLC system (LC-20, Shimadzu, Kyoto, Japan).

Insulin and its fragments (insulin chains A and B) were employed as the peptide analytes, and their interactions with the copolymer brushes were investigated. The properties of insulin are summarized in Table S2. Insulin (2.6 mg), insulin chain A (2.6 mg), and insulin chain B (2.6 mg) were each dissolved in 6 mL of 50 mM hydrochloric acid. Thereafter, the peptide solutions were filtered through a syringe filter with a 0.20-μm pore diameter. A phosphate buffer solution (pH: 7.0, 66.7 mmol/L) was supplied at 1.0 mL/min, and the elution behaviors of insulin and its fragments were observed at 280 nm.

γ-Globulin and albumin were employed as the protein analytes, and their interactions with the copolymer brushes were investigated. The properties of the adenosine nucleotides are tabulated in Table S3. γ-Globulin (2.4 mg) and albumin (2.4 mg) were each dissolved in 6 mL of a phosphate buffer solution (pH: 7.0; 66.7 mmol/L). Thereafter, the protein solutions were filtered through a syringe filter with a 0.20-μm pore diameter. A phosphate buffer solution (pH: 7.0, 66.7 mmol/L) was supplied at 1.0 mL/min, and the elution behaviors of the proteins were observed at 280 nm.

To investigate the retention behavior on the column, van't Hoff plots were obtained for the analytes [115]. The retention factor, *k′*, was obtained using Eq. (3) as follows:

(3) $$k^{\prime }=\frac{t_{R}-t_{0}}{t_{0}}$$

where *t*_*R*_ is the retention time of the analyte and *t*_*0*_ is the retention time of uracil as an initial standard.

## 3 Results and discussion

### 3.1 Characterization of the prepared copolymer-brush-grafted silica beads

The prepared copolymer-brush-grafted silica beads were characterized via CHN elemental analysis (Table 1). The CPTMS-modified silica beads displayed a significantly increased carbon content compared with the unaltered silica beads. The results showed that the silica beads were effectively modified using CPTMS via the silanization reaction. The amount of the modified CPTMS was 3.86 μmol/m^2^, which is similar to the silica-bead silanol group density. Moreover, in previous studies, the amount of CPTMS on the silica beads was ∼4.0 μmol/m^2^ [116,117], which is similar amount to that in this study. Thus, CPTMS was successfully modified on the silica beads through the reaction with their constituent silanol group.

**Table 1:** Characterization of the prepared thermoresponsive-cationic-block-copolymer-brush-modified silica beads.

Sample	Elemental composition (%) a)			Theoretical carbon composition (%)b)	Initiator density (μmol/m^2^) c)	Grafted polymer (mg/m^2^) c)
	Carbon	Hydrogen	Nitrogen			
Unmodified silica beads	0.54 ± 0.04	0.40 ± 0.44	0.33 ± 0.09	–		
CPTMS-modified beads	4.38 ± 0.08	0.33 ± 0.43	0.17 ± 0.02	47.1	3.86	
NI-QA5-tB20	17.39 ± 0.16	1.53 ± 0.62	2.90 ± 0.09	64.1		2.83
NI-QA5-nB10	19.66 ± 0.05	1.74 ± 0.09	2.45 ± 0.08	63.9		3.52

a Determined via elemental analysis. Data are the mean values with standard deviation (n = 3).

b Calculated carbon composition of the molecules.

c Calculated from the carbon composition.

The carbon compositions of the copolymer-brush-grafted silica beads NI-QA5-tB20 and NI-QA5-nB10 were determined to be considerably higher than those of the CPTMS-modified silica beads. These results confirmed that the copolymer brushes were successfully grafted onto the CPTMS-modified silica beads via ATRP. The estimated amounts of the modified copolymers of NI-QA5-tB20 and NI-QA5-nB10 were 2.83 and 3.52 mg/m^2^, respectively. These values are similar to those reported for the PNIPAAm homopolymer-brush-grafted silica beads in a previous study [118]. Thus, the copolymerizations of NIPAAm, APTAC, and tBAAm and the copolymerizations of NIPAAm, APTAC, and nBMA were successfully performed under ATRP conditions in this study. The amount of the grafted NI-QA5-nB10 was slightly higher than that of NI-QA5-tB20, probably owing to the higher reactivity of methacrylate than that of acrylamide in the ATRP process. In the polymerization of NI-QA5-tB20, all the acrylamide monomers NIPAAm, APTAC, and tBAAm were polymerized. However, for NI-QA5-nB10, the methacrylate monomer nBMA was polymerized in addition to NIPAAm and APTAC. Thus, the relatively higher reactivity of nBMA in ATRP accounts for the relatively large amount of the grafted copolymer on the silica beads.

In copolymerization, nBMA has a relatively higher reactivity than NIPAAm and APTAC. Therefore, the incorporated composition of NIPAAm and APTAC in the copolymer would be lower than the feed monomer ratio of NIPAAm and APTAC, leading to slightly reduced thermo-responsivity and cationic properties of the copolymer.

The phase transition temperature of the copolymer is crucial for temperature-responsive property change. The phase transition temperature of the PNIPAAm homopolymer in aqueous solution is ∼32 °C. It is changed by incorporating hydrophilic or hydrophobic monomers because the incorporated hydrophilic or hydrophobic monomers into PNIPAAm provide hydrophilic or hydrophobic property, respectively [119]. Furthermore, previous report indicated that the graft configuration of PNIPAAm effects on the phase transition temperature [120]. Moreover, another study on the PNIPAAm brush characterization using the surface plasmon resonance demonstrated that dense PNIPAAm brush exhibited broad phase transition from 10 °C to 40 °C [107]. Thus, we tried to measure the phase transition temperature of the prepared copolymer-brush-grafted silica beads by observing the transmittance change of the beads suspension by changing temperature. However, the copolymer-brush-grafted silica beads settled in the suspension immediately, leading to the incorrect measurement of the phase transition temperature of the grafted polymer on silica beads. On the contrary, previous report indicated that the phase transition temperature of unbound P(NIPAAm-co-APTAC-co-tBAAm) was 22.9 °C [121]. However, the phase transition temperature of the copolymer in solution was slightly different from that of the grafted copolymer brush on the silica beads. For precise measurement of the phase transition temperature of the grafted copolymer brush on silica beads, a different measurement system is required. For example, the copolymer brush was grafted onto a flat glass or silicon wafer and subsequent measurement of the water contact angle or surface roughness as a function of temperature.

### 3.2 Interaction of the copolymer brushes with adenosine nucleotides

The interaction of the prepared copolymer brushes with biomolecules was analyzed using chromatographic techniques, with the prepared copolymer-brush-modified silica beads employed as the packing materials in the HPLC column.

**Fig. 2:**
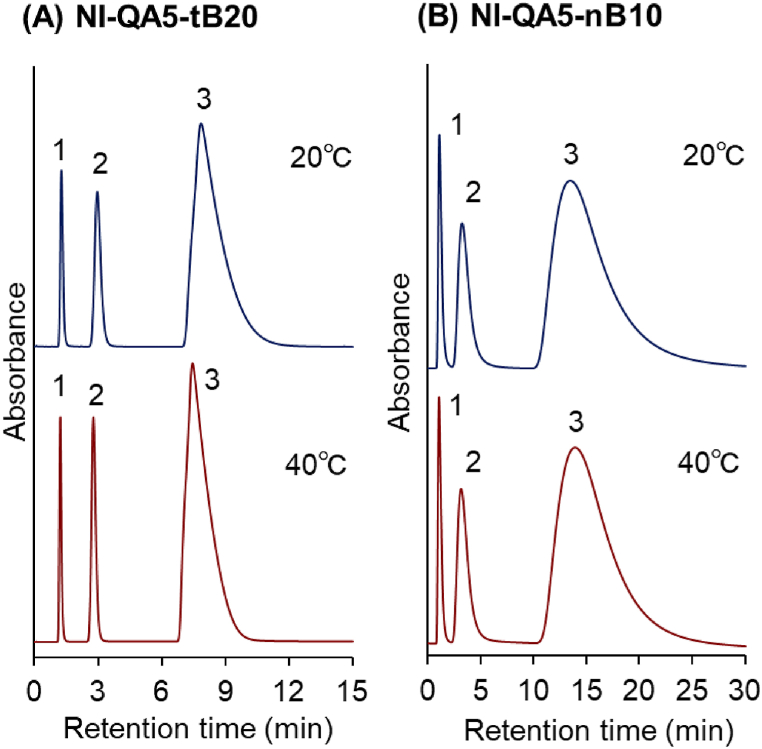
Chromatogram of the adenosine nucleotides from the thermoresponsive-copolymer-brush-grafted silica beads. (A) P(NIPAAm-*co*-APTAC-*co*-tBAAm)-brush-grafted silica-bead-packed column (NI-QA5-tB20), (B) P(NIPAAm-*co*-APTAC-*co*-nBMA)-brush-grafted silica-bead-packed column (NI-QA5-nB10). Peaks 1: AMP, 2: ADP, 3: ATP. The mobile phase is a 66.7 mM phosphate-buffered solution.

Initially, small acidic biomolecules, i.e., adenosine nucleotides, were employed as the analytes. The elution behaviors of the mixture comprising AMP, ADP, and ATP were observed at 20 °C and 40 °C (Fig. 2 A and B). Additionally, van't Hoff plot for analyzing adenosine nucleotide retention with temperature was obtained (Fig. S1). The three adenosine nucleotides were retained in the column, and the retention time increased in the order AMP < ADP < ATP. The results revealed that the retention of the adenosine nucleotides in the column was due to the electrostatic interactions between the copolymer brushes and adenosine nucleotides. AMP, ADP, and ATP have one, two, and three phosphate groups, respectively. Thus, the strengths of the anionic properties of the adenosine nucleotides are in the order AMP < ADP < ATP. Accordingly, the electrostatic interactions between the copolymer brushes and adenosine nucleotides increased in the order AMP < ADP < ATP, accounting for the difference in the retention times of the adenosine nucleotides.

The retention times of the adenosine nucleotides did not change when the column temperature was varied between 20 °C and 40 °C. In previous studies, thermoresponsive cationic copolymers containing tertiary-amine-group-modified silica beads were investigated as ion-exchange column-chromatography matrices [122]. The retention times of the adenosine nucleotides decreased with increasing column temperature, attributed to the deprotonation of the tertiary amine group in the copolymer, which induced the hydration of the copolymer [122]. Conversely, quaternary amine was introduced into the thermoresponsive copolymer in this study. Generally, quaternary amines always protonate and do not deprotonate. Thus, in this study, NI-QA5-tB20 and NI-QA5-nB10 did not deprotonate with increasing column temperature, accounting for the unchanged retention times of the adenosine nucleotides.

We assumed that the retention time of the adenosine nucleotides would be the same for both columns because the cationic monomer APTAC composition in the polymer brush was the same and electrostatic interaction between APTAC and adenosine nucleotide was almost the same. However, slightly longer retention times and wider peaks were observed for NI-QA5-nB10 compared with those for NI-QA5-tB20. This is attributed to the slightly larger amount of the grafted polymer on NI-QA5-nB10. A longer copolymer was grafted on NI-QA5-nB10 compared with that on NI-QA5-tB20, as evidenced by the result of the CHN elemental analysis. In addition, previous studies have shown that longer-polymer-brush-grafted silica beads exhibit longer retention times and wider peaks because the analytes tend to diffuse into the layer of the longer polymer brush [123]. Thus, NI-QA5-nB10 exhibited slightly longer retention times and wider peaks of the adenosine nucleotides than NI-QA5-tB20.

### 3.3 Interaction of the copolymer brushes and peptides

**Fig. 3:**
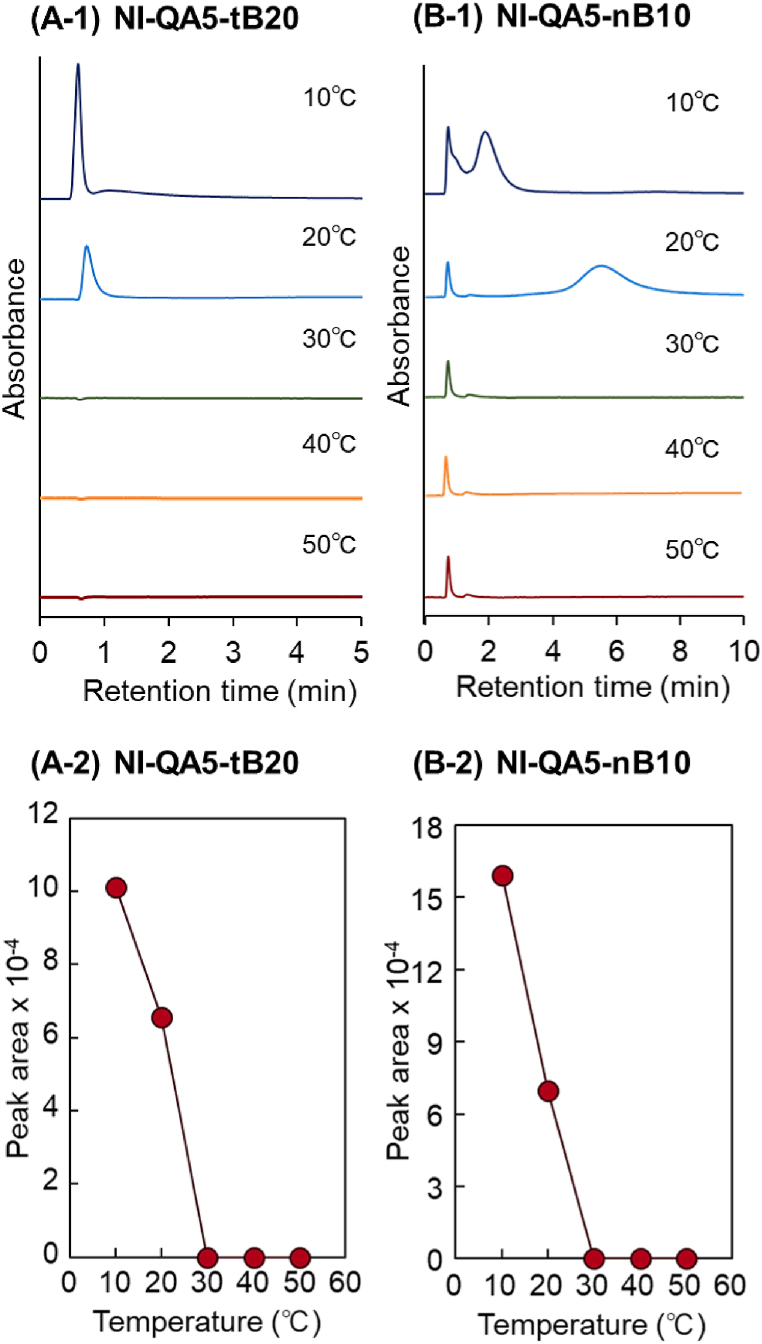
Temperature-dependent elution behavior of the insulin from the prepared bead–packed column. (A) P(NIPAAm-*co*-APTAC-*co*-tBAAm) brush–grafted silica bead–packed column (NI-QA5-tB20), (B) P(NIPAAm-*co*-APTAC-*co*-nBMA) brush–grafted silica bead–packed column (NI-QA5-nB10).

**Fig. 4:**
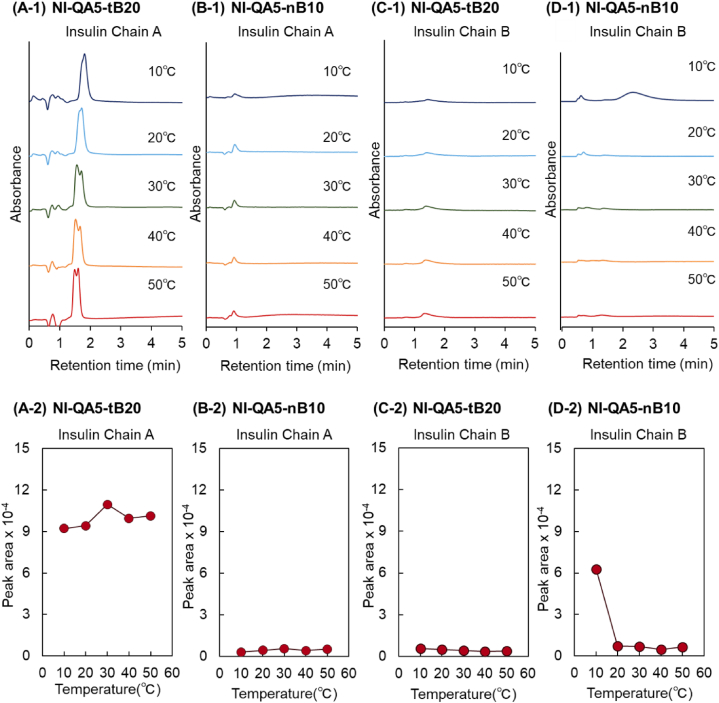
Temperature-dependent elution behavior of insulin chain A (A) and (C) and chain B (B) and (D) from the prepared-bead-packed column. (A) P(NIPAAm-*co*-APTAC-*co*-tBAAm) brush–grafted silica bead–packed column (NI-QA5-tB20), (B) P(NIPAAm-*co*-APTAC-*co*-BMA) brush–grafted silica bead–packed column (NI-QA5-nB10).

To investigate the interaction between the copolymer brush and peptides, the elution behaviors of insulin and its fragment were observed (Fig. 3, Fig. 4). Furthermore, van't Hoff plot for analyzing peptide retention with temperature was obtained (Fig. S2). Initially, the elution behavior of insulin in the bead-packed column was observed (Fig. 3 A and B). In NI-QA5-tB20 and NI-QA5-nB10 columns, insulin was eluted at low temperatures, e.g., 10 °C and 20 °C. Conversely, insulin was not eluted in the columns at high temperatures. This is attributable to the adsorption of insulin in the column through a combination of hydrophobic and electrostatic interactions between the copolymer brushes and insulin. In previous studies, a P(NIPAAm-*co*-tBAAm) brush–modified poly(hydroxy methacrylate) bead–packed column exhibited increased insulin retention times as the column temperature increased, which was attributed to the increased hydrophobic interaction between the dehydrated P(NIPAAm-*co*-tBAAm) and insulin [124]. In addition, P(NIPAAm-*co*-BMA) brush–modified silica bead–packed columns exhibited increased insulin retention times, which was attributed to the enhanced hydrophobic interaction between the dehydrated P(NIPAAm-*co*-BMA) and insulin [112]. Similarly, in this study, both the NI-QA5-tB20 and NI-QA5-nB10 columns underwent hydrophobic interactions with insulin.

Furthermore, the electrostatic interaction of these copolymers with insulin was possible because these copolymers have strong cationic groups and insulin has a negative charge in a neutral buffer environment (pH: 7.0). Based on the elution behavior of the adenosine nucleotides, we established that the cationic property of the copolymer brushes did not change when the column temperature was changed (Fig. 2). Thus, the copolymer brushes underwent electrostatic interactions with insulin at low and high temperatures.

The hydrophobic and electrostatic interactions of the copolymer brushes and insulin progress at low and high temperatures as follows: at low temperatures, only electrostatic interactions occur because the copolymers are hydrated and the hydrophobic interaction is weak. Thus, insulin is not adsorbed on the copolymer brushes. Conversely, at high temperatures, the copolymer brushes are dehydrated and become hydrophobic, enabling their interaction with insulin. Simultaneously, electrostatic interaction occurs between the copolymer brushes and insulin because the cationic property of the copolymer brushes is retained at high temperatures. Thus, both hydrophobic and electrostatic interactions occur between the copolymer brush and insulin at high temperatures, leading to the adsorption of insulin on the copolymer brush.

Insulin structure seemed to be changed by increasing temperature. However, previous reports demonstrated that the structure of the peptide does not change as the temperature increases from 10 °C to 40 °C [125]. Thus, the peptide adsorption on the copolymer brush was mainly attributed to the interaction between the copolymer and insulin rather than the structural change of insulin.

Furthermore, previous reports demonstrated that the adsorbed peptides and protein on the ionic thermoresponsive polymer on silica beads at high column temperature were desorbed by reducing temperature [[45], [46], [47], [48],^54^,^114^,^121^,^126^]. Thus, in a similar manner, the adsorbed insulin can be recovered by reducing column temperature.

The elution behaviors of insulin chains A and B were observed (Fig. 4A–D). Insulin chain A was eluted in the NI-QA5-tB20 column at all temperatures. In previous studies, P(NIPAAm-*co*-tBAAm)-brush- and P(NIPAAm-*co*-tBAAm)-brush-modified bead-packed columns scarcely retained insulin chain A. This is probably because insulin chain A is relatively hydrophilic and the hydrophobic interaction of the copolymers with insulin chain A is not sufficient for the retention of insulin chain A [112,124]. Similarly, insulin chain A was eluted from the NI-QA5-tB20 column, although additional electrostatic interactions occurred with insulin chain A. Conversely, insulin chain A adsorbed on NI-QA5-nB10 at all temperatures, probably because of the strong hydrophobicity of nBMA. In a previous study, P(NIPAAm-*co*-2-acrylamido-2-methylpropanesulfonic acid (AMPS)-*co*-tBAAm)-brush- and P(NIPAAm-*co*-AMPS*-co*-nBMA)-brush-grafted silica beads were prepared as chromatographic matrices for antibody purification, and the antibody-adsorption performances of the prepared copolymer brushes were investigated [48]. The P(NIPAAm-*co*-AMPS*-co*-nBMA) brush exhibited a higher antibody-adsorption capacity than the P(NIPAAm-*co*-AMPS*-co*-tBAAm) brush, which was attributed to the hydrophobicity of the incorporated nBMA [48]. Similarly, NI-QA5-nB10 exhibits a stronger adsorption capacity for insulin chain A than NI-QA5-tB20.

Insulin chain B adsorbed on the copolymer-brush-grafted silica-bead-packed columns. In previous studies, the P(NIPAAm-*co*-tBAAm)-brush- and P(NIPAAm-*co*-tBAAm)-brush-modified silica-bead-packed columns retained insulin chain B because of its higher hydrophobicity compared with that of insulin chain A [112,124]. Similarly, insulin chain B adsorbed on the copolymer brush through hydrophobic and electrostatic interactions.

### 3.4 Interaction of the copolymer brushes with proteins

**Fig. 5:**
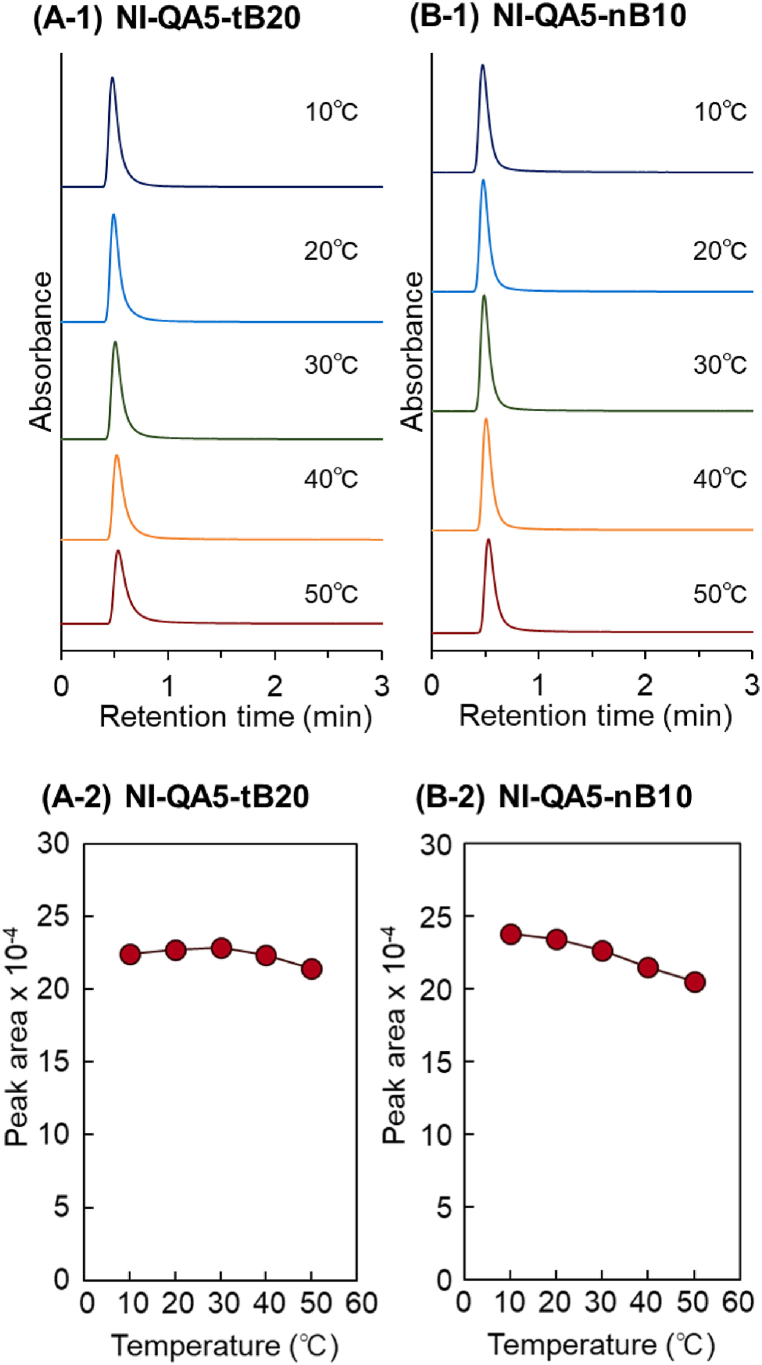
Temperature-dependent elution behavior of γ-globulin from the prepared-bead-packed column. (A) P(NIPAAm-*co*-APTAC-*co*-tBAAm) brush–grafted silica bead–packed column (NI-QA5-tB20), (B) P(NIPAAm-*co*-APTAC-*co*-BMA) brush–grafted silica bead–packed column (NI-QA5-nB10).

**Fig. 6:**
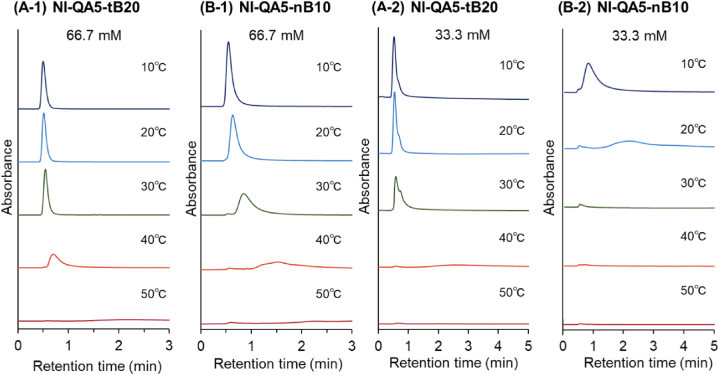
Temperature-dependent elution behavior of albumin from the prepared-bead-packed column. (A) P(NIPAAm-*co*-APTAC-*co*-tBAAm) brush–grafted silica bead–packed column (NI-QA5-tB20) and (B) P(NIPAAm-*co*-APTAC-*co*-BMA) brush–grafted silica bead–packed column (NI-QA5-nB10). The mobile phase is a (1) 66.7 mM phosphate-buffered solution (pH: 7.0) or a (2) 33.3 mM phosphate-buffered solution (pH: 7.0).

**Fig. 7:**
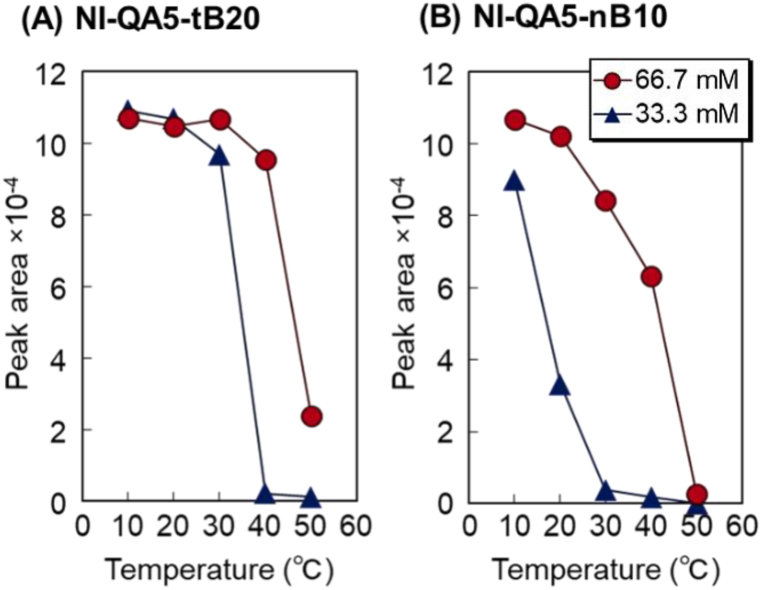
Peak area of the eluted albumin from the prepared-bead-packed column. (A) P(NIPAAm-*co*-APTAC-*co*-tBAAm) brush–grafted silica bead–packed column (NI-QA5-tB20) and (B) P(NIPAAm-*co*-APTAC-*co*-BMA) brush–grafted silica bead–packed column (NI-QA5-nB10).

To investigate the interaction of the copolymer brush with proteins, the elution behaviors of globulin and albumin were observed (Fig. 5, Fig. 6, Fig. 7). Furthermore, van't Hoff plot for analyzing protein retention with temperature was obtained (Fig. S3). First, the elution behavior of γ-globulin was investigated (Fig. 5). The NI-QA5-tB20 and NI-QA5-nB10 columns eluted γ-globulin at short retention times at all temperatures (Fig. 5 A-1 and B-1). γ-Globulin was eluted with a shorter retention time compared to t_0_, and a negative value of the retention factor was observed (Fig. S3). This is due to the large molecular size of the γ-globulin. Previous studies investigated the calibration curves of similar types of PNIPAAm-modified silica beads packed column using glucose and pullulan standards [127,128]. The larger size of the pullulan standard with the similar molecular size of γ-globulin exhibited a smaller retention volume from the PNIPAAm-modified beads packed column than that from the unmodified beads packed column, indicating that larger molecules did not enter into the pore of the PNIPAAm-modified beads. Similarly, γ-globulin did not enter into the pores of the beads of NI-QA5-tB20 and NI-QA5-nB10 columns.

The eluted peak area of γ-globulin was almost the same at all temperatures (Fig. 5 A-2 and B-2). Results indicated that γ-globulin was not retained and adsorbed on the NI-QA5-tB20 and NI-QA5-nB10 columns, which is attributed to its weak interactions with the copolymer brushes. γ-Globulin exhibits a relatively neutral property (isoelectric point = 6.85). Thus, the electrostatic interaction of the copolymer brush with γ-globulin was weak. In previous studies, the proteins did not adsorb on the P(NIPAAm-*co*-tBAAm) and P(NIPAAm-*co*-nBMA) brushes through only hydrophobic interactions [45,46]. Similarly, γ-globulin did not adsorb on the NI-QA5-tB20 and NI-QA5-nB10 copolymer brushes through only hydrophobic interactions.

The elution behavior of albumin in the NI-QA5-tB20 and NI-QA5-nB10 columns was studied (Fig. 6, Fig. 7). At low temperatures (e.g., 10 °C and 20 °C), albumin was eluted from the NI-QA5-tB20 and NI-QA5-nB10 columns (Fig. 6 A-1 and B-1). This result indicated that the interaction of the copolymer brushes with albumin was weak. At 30 °C, albumin was eluted with a short retention time in the NI-QA5-tB20 column, whereas for the NI-QA5-nB10 column, the retention time of albumin increased and a slight amount of albumin was adsorbed (Fig. 6 A-1 and 6B-1, Fig. 7 A and B). This result indicated that albumin interacted with the NI-QA5-nB10 copolymer brushes through hydrophobic and electrostatic interactions. At 40 °C, a small amount of albumin was absorbed on the NI-QA5-tB20 copolymer brush column, whereas half of that amount was adsorbed on the NI-QA5-nB10 copolymer brush. At 50 °C, most of the albumin adsorbed in the NI-QA5-tB20 and NI-QA5-nB10 columns (Fig. 6 A-1 and 6B-1, Fig. 7 A and B). The results indicated that albumin interacted with the copolymer brush through hydrophobic and electrostatic interactions, resulting in its adsorption on the copolymer brushes. Moreover, the NI-QA5-nB10 column exhibited a higher albumin-adsorption efficiency than the NI-QA5-tB20 column.

To investigate the role of the electrostatic interaction between albumin and the copolymer brushes in albumin adsorption, the mobile phase was changed from a 66.7 to a 33.3 mM phosphate-buffered solution (pH: 7.0) and the electrostatic interaction between the copolymer brushes and albumin was enhanced (Fig. 6 A-2 and 6B-2, Fig. 7 A and B). Albumin was adsorbed on the NI-QA5-nB10 columns even at a relatively low temperature of 20 °C. This is because of the enhanced electrostatic interaction between albumin and the copolymer brushes at a reduced concentration of the phosphate-buffered solution (the mobile phase). At 40 °C, most of the albumin had adsorbed on the NI-QA5-tB20 and NI-QA5-nB10 copolymer brushes. This was attributed to the hydrophobic and enhanced electrostatic interactions. The peak area of the albumin-adsorption graph indicated that albumin tended to adsorb when the concentration of the phosphate-buffered solution was 33.3 mM (Fig. 7). The results also indicated that at the low phosphate buffer concentration of 33.3 mM, albumin adsorbs on the copolymer brushes even at low temperatures, unlike the case at 66.7 mM phosphate buffer concentration (Fig. 7).

By comparing NI-QA5-tB20 and NI-QA5-nB10 columns, NI-QA5-nB10 columns exhibited albumin adsorption even at low temperatures. This is probably due to the higher hydrophobicity of nBMA than tBAAm. However, previous reports showed that the thermoresponsive ionic copolymer with nBMA exhibited effective protein adsorption compared to that with tBAAm, which is attributed to the enhanced hydrophobic interaction [48]. Similarly, NI-QA5-nB10 exhibited effective adsorption of albumin.

The albumin structure seemed to be changed by increasing temperature. However, previous reports showed that the structure of the albumin does not change as the temperature increases from 10 °C to 50 °C [125]. Thus, the albumin adsorption on the copolymer brush was mainly attributed to the interaction between the copolymer and albumin rather than the structural change of albumin.

The results indicated that the developed thermoresponsive strong cationic copolymer brushes with different hydrophobic groups can interact with peptides and proteins through a combination of electrostatic and temperature-modulated hydrophobic interactions. Thus, the developed copolymer brush holds potential for application in chromatographic matrices for peptide analysis and protein purification.

## 4 Conclusions

We developed thermoresponsive strong cationic copolymer brushes with different hydrophobic groups (P(NIPAAm-*co*-APTAC*-co*-nBMA) and P(NIPAAm-*co*-APTAC*-co*-tBAAm)) on the silica beads through ATRP. The results of CHN elemental analysis of the copolymer-brush-grafted beads revealed that a considerable amount of the copolymer was grafted onto the silica beads owing to the ATRP reaction in this study. The electrostatic and hydrophobic interactions of the prepared copolymer brushes and biomolecules were analyzed via HPLC using the copolymer brush–modified bead-packed columns. Adenosine nucleotides were retained in the prepared columns. The retention time increased in the order AMP < ADP < ATP, which was attributed to the electrostatic interaction of the copolymer brush and adenosine nucleotides. The retention time of adenosine nucleotides did not change when the column temperature was changed, indicating that the cationic property of the copolymer brush was not dependent on the temperature. Insulin was adsorbed on the copolymer brushes at high temperatures owing to electrostatic and hydrophobic interactions. Albumin was adsorbed on the copolymer brushes at high temperatures owing to electrostatic and hydrophobic interactions as well. Using a low concentration of a phosphate buffer solution, albumin was adsorbed on the copolymer brushes even at relatively low temperatures, which was attributed to the enhanced electrostatic interaction between the copolymer and albumin. P(NIPAAm-*co*-APTAC*-co*-nBMA) brush exhibited more effective albumin adsorption than the P(NIPAAm-*co*-APTAC*-co*-tBAAm) brush, which is attributed to the enhanced hydrophobic interaction. The results indicated that the developed thermoresponsive, strong, cationic copolymer brushes can interact with peptides and proteins through a combination of electrostatic and temperature-modulated hydrophobic interactions. Thus, the developed copolymer brush holds potential for application in chromatographic matrices for the analysis and purification of peptides and proteins.

## Data availability statement

The data of this study will be made available on request to the corresponding author.

## CRediT authorship contribution statement

**Kenichi Nagase:** Writing – review & editing, Writing – original draft, Supervision, Project administration, Methodology, Investigation, Funding acquisition, Conceptualization. **Sayaka Suzuki:** Writing – review & editing, Methodology, Investigation, Formal analysis, Data curation. **Hideko Kanazawa:** Writing – review & editing, Supervision.

## Declaration of competing interest

The authors declare the following financial interests/personal relationships which may be considered as potential competing interests:

Kenichi Nagase is an Associated Editor of Heliyon.

## References

[1] Stuart M.A.C., Huck W.T.S., Genzer J., Muller M., Ober C., Stamm M., Sukhorukov G.B., Szleifer I., Tsukruk V.V., Urban M., Winnik F., Zauscher S., Luzinov I., Minko S. (2010). Emerging applications of stimuli-responsive polymer materials. Nat. Mater..

[2] Hoffman A.S., Stayton P.S. (2007). Conjugates of stimuli-responsive polymers and proteins. Prog. Polym. Sci..

[3] Liu F., Urban M.W. (2010). Recent advances and challenges in designing stimuli-responsive polymers. Prog. Polym. Sci..

[4] Mano J.F. (2008). Stimuli-responsive polymeric systems for biomedical applications. Adv. Eng. Mater..

[5] Gil E.S., Hudson S.M. (2004). Stimuli-reponsive polymers and their bioconjugates. Prog. Polym. Sci..

[6] Roy D., Brooks W.L.A., Sumerlin B.S. (2013). New directions in thermoresponsive polymers. Chem. Soc. Rev..

[7] Heskins M., Guillet J.E. (1968). Solution properties of poly(*N*-isopropylacrylamide). J. Macromol. Sci..

[8] Schild H.G. (1992). Poly(*N*-isopropylacrylamide): experiment, theory and application. Prog. Polym. Sci..

[9] Halperin A., Kröger M., Winnik F.M. (2015). Poly(*N*-isopropylacrylamide) phase diagrams: fifty years of research. Angew. Chem. Int. Ed..

[10] Tang L., Wang L., Yang X., Feng Y., Li Y., Feng W. (2021). Poly(*N*-isopropylacrylamide)-based smart hydrogels: design, properties and applications. Prog. Mater. Sci..

[11] Nagase K., Onuma T., Yamato M., Takeda N., Okano T. (2015). Enhanced wettability changes by synergistic effect of micro/nanoimprinted substrates and grafted thermoresponsive polymer brushes. Macromol. Rapid Commun..

[12] Nagase K., Matsuda J., Takeuchi A., Ikemoto Y. (2023). Hydration and dehydration behaviors of poly(N-isopropylacrylamide)-grafted silica beads. Surface. Interfac..

[13] Shaibie N.A., Ramli N.A., Mohammad Faizal N.D.F., Srichana T., Mohd Amin M.C.I. (2023). Poly(N-isopropylacrylamide)-Based polymers: recent overview for the development of temperature-responsive drug delivery and biomedical applications. Macromol. Chem. Phys..

[14] Chung J.E., Yokoyama M., Yamato M., Aoyagi T., Sakurai Y., Okano T. (1999). Thermo-responsive drug delivery from polymeric micelles constructed using block copolymers of poly(*N*-isopropylacrylamide) and poly(butylmethacrylate). J. Contr. Release.

[15] Nakayama M., Okano T. (2011). Multi-targeting cancer chemotherapy using temperature-responsive drug carrier systems. React. Funct. Polym..

[16] Wei H., Cheng S.-X., Zhang X.-Z., Zhuo R.-X. (2009). Thermo-sensitive polymeric micelles based on poly(*N*-isopropylacrylamide) as drug carriers. Prog. Polym. Sci..

[17] Nemoto R., Fujieda K., Hiruta Y., Hishida M., Ayano E., Maitani Y., Nagase K., Kanazawa H. (2019). Liposomes with temperature-responsive reversible surface properties. Colloids Surf., B.

[18] Han H., Zhao X., Ma H., Zhang Y., Lei B. (2023). Multifunctional injectable hydrogels with controlled delivery of bioactive factors for efficient repair of intervertebral disc degeneration. Heliyon.

[19] Arjun P., Freeman J.L., Kannan R.R. (2022). Neurospecific fabrication and toxicity assessment of a PNIPAM nanogel encapsulated with trans-tephrostachin for blood-brain-barrier permeability in zebrafish model. Heliyon.

[20] Ajith S., Almomani F., Elhissi A., Husseini G.A. (2023). Nanoparticle-based materials in anticancer drug delivery: current and future prospects. Heliyon.

[21] Aswathy S.H., Narendrakumar U., Manjubala I. (2020). Commercial hydrogels for biomedical applications. Heliyon.

[22] Nagase K., Hasegawa M., Ayano E., Maitani Y., Kanazawa H. (2019). Effect of polymer phase transition behavior on temperature-responsive polymer-modified liposomes for siRNA transfection. Int. J. Mol. Sci..

[23] Maekawa-Matsuura M., Fujieda K., Maekawa Y., Nishimura T., Nagase K., Kanazawa H. (2019). LAT1-Targeting thermoresponsive liposomes for effective cellular uptake by cancer cells. ACS Omega.

[24] Hiruta Y. (2022). Poly(N-isopropylacrylamide)-based temperature- and pH-responsive polymer materials for application in biomedical fields. Polym. J..

[25] Gheysoori P., Paydayesh A., Jafari M., Peidayesh H. (2023). Thermoresponsive nanocomposite hydrogels based on Gelatin/poly (N–isopropylacrylamide) (PNIPAM) for controlled drug delivery. Eur. Polym. J..

[26] Rezk A.I., Kim Y.-H., Chun S., Park C.H., Kim C.S. (2023). Thermo-responsive-polymeric-gates of poly(N-isopropylacrylamide)/N-(hydroxymethyl)acrylamide coated magnetic nanoparticles as a synergistic approach to cancer therapy: drug release and kinetics models of chemothermal magnetic nanoparticles. Mater. Des..

[27] Nagase K. (2024). Bioanalytical technologies using temperature-responsive polymers. Anal. Sci..

[28] Mori T., Maeda M. (2004). Temperature-responsive formation of colloidal nanoparticles from poly(*N*-isopropylacrylamide) grafted with single-stranded DNA. Langmuir.

[29] Ebara M., Hoffman J.M., Hoffman A.S., Stayton P.S. (2006). Switchable surface traps for injectable bead-based chromatography in PDMS microfluidic channels. Lab Chip.

[30] Islam M.R., Xie S., Huang D., Smyth K., Serpe M.J. (2015). Poly (*N*-isopropylacrylamide) microgel-based optical devices for humidity sensing. Anal. Chim. Acta.

[31] Iwasaki W., Toda H., Morita N., Motomura T., Fujio Y., Takemura K., Nakanishi Y., Nakashima Y. (2022). A thermoresponsive valve to control fluid flow in microfluidic paper-based devices. Microfluid. Nanofluidics.

[32] Chang Y.-X., Wang C.-F., Chang C.-J., Lu C.-H., Chen J.-K. (2023). Fabrication of scalable poly(N-isopropylacrylamide)/gold nanoparticles composite ring array as LSPR sensor for label-free biosensor application. Sensors Actuators B: Chem..

[33] Nagase K., Nishiyama T., Inoue M., Kanazawa H. (2021). Temperature responsive chromatography for therapeutic drug monitoring with an aqueous mobile phase. Sci. Rep..

[34] Nagase K., Inoue S., Inoue M., Kanazawa H. (2022). Two-dimensional temperature-responsive chromatography using a poly(N-isopropylacrylamide) brush-modified stationary phase for effective therapeutic drug monitoring. Sci. Rep..

[35] Nagase K., Takagi H., Nakada H., Ishikawa H., Nagata Y., Aomori T., Kanazawa H. (2022). Chromatography columns packed with thermoresponsive-cationic-polymer-modified beads for therapeutic drug monitoring. Sci. Rep..

[36] Nagase K., Watanabe M., Zen F., Kanazawa H. (2019). Temperature-responsive mixed-mode column containing temperature-responsive polymer-modified beads and anionic polymer-modified beads. Anal. Chim. Acta.

[37] Nagase K., Matsumoto K., Kanazawa H. (2022). Temperature-responsive mixed-mode column for the modulation of multiple interactions. Sci. Rep..

[38] Maekawa Y., Okamoto N., Okada Y., Nagase K., Kanazawa H. (2020). Green analytical method for the simultaneous analysis of cytochrome P450 probe substrates by poly(N-isopropylacrylamide)-based temperature-responsive chromatography. Sci. Rep..

[39] Yoshida R. (2022). Creation of softmaterials based on self-oscillating polymer gels. Polym. J..

[40] Tamate R., Mizutani Akimoto A., Yoshida R. (2016). Recent advances in self-oscillating polymer material systems. Chem. Rec..

[41] Masuda T., Akimoto A.M., Nagase K., Okano T., Yoshida R. (2015). Design of self-oscillating polymer brushes and control of the dynamic behaviors. Chem. Mater..

[42] Homma K., Masuda T., Akimoto A.M., Nagase K., Itoga K., Okano T., Yoshida R. (2017). Fabrication of micropatterned self-oscillating polymer brush for direction control of chemical waves. Small.

[43] Terefe N.S., Glagovskaia O., De Silva K., Stockmann R. (2014). Application of stimuli responsive polymers for sustainable ion exchange chromatography. Food Bioprod. Process..

[44] Nagase K., Kanazawa H. (2020). Temperature-responsive chromatography for bioseparations: a review. Anal. Chim. Acta.

[45] Nagase K., Kobayashi J., Kikuchi A., Akiyama Y., Kanazawa H., Okano T. (2016). Thermoresponsive anionic block copolymer brushes with a strongly anionic bottom segment for effective interactions with biomolecules. RSC Adv..

[46] Nagase K., Kobayashi J., Kikuchi A., Akiyama Y., Kanazawa H., Okano T. (2016). Protein separations via thermally responsive ionic block copolymer brush layers. RSC Adv..

[47] Okubo K., Ikeda K., Oaku A., Hiruta Y., Nagase K., Kanazawa H. (2018). Protein purification using solid-phase extraction on temperature-responsive hydrogel-modified silica beads. J. Chromatogr. A.

[48] Nagase K., Ishii S., Ikeda K., Yamada S., Ichikawa D., Akimoto A., Hattori Y., Kanazawa H. (2020). Antibody drug separation using thermoresponsive anionic polymer brush modified beads with optimised electrostatic and hydrophobic interactions. Sci. Rep..

[49] Nagase K., Kitazawa S., Kogure T., Yamada S., Katayama K., Kanazawa H. (2022). Viral vector purification with thermoresponsive-anionic mixed polymer brush modified beads-packed column. Sep. Purif. Technol..

[50] Nagase K., Ishii S., Takeuchi A., Kanazawa H. (2022). Temperature-modulated antibody drug separation using thermoresponsive mixed polymer brush-modified stationary phase. Sep. Purif. Technol..

[51] Nagase K., Yamazaki K., Maekawa Y., Kanazawa H. (2023). Thermoresponsive bio-affinity interfaces for temperature-modulated selective capture and release of targeted exosomes. Materials Today Bio.

[52] Mizutani A., Nagase K., Kikuchi A., Kanazawa H., Akiyama Y., Kobayashi J., Annaka M., Okano T. (2010). Effective separation of peptides using highly dense thermo-responsive polymer brush-grafted porous polystyrene beads. J. Chromatogr. B.

[53] Nagase K., Kobayashi J., Kikuchi A., Akiyama Y., Kanazawa H., Okano T. (2013). Thermally modulated cationic copolymer brush on monolithic silica rods for high-speed separation of acidic biomolecules. ACS Appl. Mater. Interfaces.

[54] Nagase K., Kobayashi J., Kikuchi A., Akiyama Y., Kanazawa H., Okano T. (2014). Thermoresponsive anionic copolymer brushes containing strong acid moieties for effective separation of basic biomolecules and proteins. Biomacromolecules.

[55] Maekawa Y., Ayano E., Nagase K., Kanazawa H. (2021). Effective separation for new therapeutic modalities utilizing temperature-responsive chromatography. Anal. Sci..

[56] Nagase K., Sakurada Y., Onizuka S., Iwata T., Yamato M., Takeda N., Okano T. (2017). Thermoresponsive polymer-modified microfibers for cell separations. Acta Biomater..

[57] Nagase K., Shukuwa R., Onuma T., Yamato M., Takeda N., Okano T. (2017). Micro/nano-imprinted substrates grafted with a thermoresponsive polymer for thermally modulated cell separation. J. Mater. Chem. B.

[58] Nagase K., Ota A., Hirotani T., Yamada S., Akimoto A.M., Kanazawa H. (2020). Thermoresponsive cationic block copolymer brushes for temperature-modulated stem cell separation. Macromol. Rapid Commun..

[59] Sung T.-C., Su H.C., Ling Q.-D., Kumar S.S., Chang Y., Hsu S.-T., Higuchi A. (2020). Efficient differentiation of human pluripotent stem cells into cardiomyocytes on cell sorting thermoresponsive surface. Biomaterials.

[60] Nagase K., Uchikawa N., Hirotani T., Akimoto A.M., Kanazawa H. (2020). Thermoresponsive anionic copolymer brush-grafted surfaces for cell separation. Colloids Surf., B.

[61] Nagase K., Kojima N., Goto M., Akaike T., Kanazawa H. (2022). Thermoresponsive block copolymer brush for temperature-modulated hepatocyte separation. J. Mater. Chem. B.

[62] Nagase K., Wakayama H., Matsuda J., Kojima N., Kanazawa H. (2023). Thermoresponsive mixed polymer brush to effectively control the adhesion and separation of stem cells by altering temperature. Materials Today Bio.

[63] Guerron A., Phan H.T., Peñaloza-Arias C., Brambilla D., Roullin V.G., Giasson S. (2022). Selectively triggered cell detachment from poly(N-isopropylacrylamide) microgel functionalized substrates. Colloids Surf., B.

[64] Nagase K., Shukuwa R., Takahashi H., Takeda N., Okano T. (2020). Enhanced mechanical properties and cell separation with thermal control of PIPAAm-brushed polymer-blend microfibers. J. Mater. Chem. B.

[65] Nagase K., Shimura M., Shimane R., Hanaya K., Yamada S., Akimoto A.M., Sugai T., Kanazawa H. (2021). Selective capture and non-invasive release of cells using a thermoresponsive polymer brush with affinity peptides. Biomater. Sci..

[66] Nagase K., Edatsune G., Nagata Y., Matsuda J., Ichikawa D., Yamada S., Hattori Y., Kanazawa H. (2021). Thermally-modulated cell separation columns using a thermoresponsive block copolymer brush as a packing material for the purification of mesenchymal stem cells. Biomater. Sci..

[67] Kuno G., Imaizumi Y., Matsumoto A. (2022). Application of the water-insoluble, temperature-responsive block polymer poly(butyl methacrylate-block-N-isopropylacrylamide) for pluripotent stem cell culture and cell-selective detachment. J. Biosci. Bioeng..

[68] Nagase K., Mukae N., Kikuchi A., Okano T. (2012). Thermally modulated retention of lymphocytes on polymer-brush-grafted glass beads. Macromol. Biosci..

[69] Nagase K., Okada A., Matsuda J., Ichikawa D., Hattori Y., Kanazawa H. (2022). A thermoresponsive cationic block copolymer brush-grafted silica bead interface for temperature-modulated separation of adipose-derived stem cells. Colloids Surf., B.

[70] Lanzalaco S., Mingot J., Torras J., Alemán C., Armelin E. (2023). Recent advances in poly(N-isopropylacrylamide) hydrogels and derivatives as promising materials for biomedical and engineering emerging applications. Adv. Eng. Mater..

[71] Hirotani T., Nagase K. (2024). Temperature-modulated separation of vascular cells using thermoresponsive-anionic block copolymer-modified glass. Regenerative Therapy.

[72] Yamada N., Okano T., Sakai H., Karikusa F., Sawasaki Y., Sakurai Y. (1990). Thermo-responsive polymeric surfaces; control of attachment and detachment of cultured cells. Makromol. Chem., Rapid Commun..

[73] Akiyama Y., Kikuchi A., Yamato M., Okano T. (2004). Ultrathin poly(N-isopropylacrylamide) grafted layer on polystyrene surfaces for cell adhesion/detachment control. Langmuir.

[74] Takahashi H., Nakayama M., Yamato M., Okano T. (2010). Controlled chain length and graft density of thermoresponsive polymer brushes for optimizing cell sheet harvest. Biomacromolecules.

[75] Akiyama Y. (2021). Influence of poly(*N*-isopropylacrylamide) (PIPAAm) graft density on properties of PIPAAm grafted poly(dimethylsiloxane) surfaces and their stability. Heliyon.

[76] Capella V., Rivero R.E., Liaudat A.C., Ibarra L.E., Roma D.A., Alustiza F., Mañas F., Barbero C.A., Bosch P., Rivarola C.R., Rodriguez N. (2019). Cytotoxicity and bioadhesive properties of poly-N-isopropylacrylamide hydrogel. Heliyon.

[77] Akimoto A., Niitsu E., Nagase K., Okano T., Kanazawa H., Yoshida R. (2018). Mesenchylmal stem cell culture on poly(*N*-isopropylacrylamide) hydrogel with repeated thermo-stimulation. Int. J. Mol. Sci..

[78] Nakao M., Kim K., Nagase K., Grainger D.W., Kanazawa H., Okano T. (2019). Phenotypic traits of mesenchymal stem cell sheets fabricated by temperature-responsive cell culture plate: structural characteristics of MSC sheets. Stem Cell Res. Ther..

[79] Nakao M., Inanaga D., Nagase K., Kanazawa H. (2019). Characteristic differences of cell sheets composed of mesenchymal stem cells with different tissue origins. Regenerative Therapy.

[80] Nakao M., Matsui M., Kim K., Nishiyama N., Grainger D.W., Okano T., Kanazawa H., Nagase K. (2023). Umbilical cord-derived mesenchymal stem cell sheets transplanted subcutaneously enhance cell retention and survival more than dissociated stem cell injections. Stem Cell Res. Ther..

[81] Nakayama M., Toyoshima Y., Kikuchi A., Okano T. (2021). Micropatterned smart culture surfaces via multi-step physical coating of functional block copolymers for harvesting cell sheets with controlled sizes and shapes. Macromol. Biosci..

[82] Kobayashi J., Okano T. (2023). Reproducible preparation of primary rat hepatocyte sheets using a thermoresponsive culture dish. Tissue Eng. C.

[83] Nagase K., Nagaoka M., Nakano Y., Utoh R. (2024). bFGF-releasing biodegradable nanoparticles for effectively engrafting transplanted hepatocyte sheet. J. Contr. Release.

[84] Yang L., Fan X., Zhang J., Ju J. (2020). Preparation and characterization of thermoresponsive poly(N-isopropylacrylamide) for cell culture applications. Polymers.

[85] Ahmadi S., Nasiri M., Pourrajab-miandoab A., Jafari A. (2023). Temperature responsive poly(N-isopropylacrylamide-co-styrene) nanofilms for non-enzymatic cell sheet harvesting. Prog. Org. Coating.

[86] Hajibabazadeh S., Ghaleh H., Abbasi F., Foroutani K. (2023). Design of thermo-responsive cell culture dishes using poly(N-isopropylacrylamide)-block-polystyrene copolymers for cell sheet technology. Eur. Polym. J..

[87] Nakayama M., Kanno T., Takahashi H., Kikuchi A., Yamato M., Okano T. (2021). Terminal cationization of poly(N-isopropylacrylamide) brush surfaces facilitates efficient thermoresponsive control of cell adhesion and detachment. Sci. Technol. Adv. Mater..

[88] Nakayama M., Toyoshima Y., Chinen H., Kikuchi A., Yamato M., Okano T. (2020). Water stable nanocoatings of poly(N-isopropylacrylamide)-based block copolymers on culture insert membranes for temperature-controlled cell adhesion. J. Mater. Chem. B.

[89] Yang L., Ren P., Wei D., Liang M., Xu L., Tao Y., Jiao G., Zhang T., Zhang Q. (2024). Temperature-Controlled screening of catechol groups in poly(N-Isopropylacrylamide-co-Dopamine methacrylamide) for cell detachment. ACS Appl. Polym. Mater..

[90] Nakao M., Nagase K. (2024). Harvesting methods of umbilical cord-derived mesenchymal stem cells from culture modulate cell properties and functions. Regenerative Therapy.

[91] Nagase K., Nagaoka M., Matsuda J., Kojima N. (2024). Thermoresponsive block-copolymer brush-modified interfaces for effective fabrication of hepatocyte sheets. Mater. Des..

[92] Dutta S., Shreyash N., Satapathy B.K., Saha S. (2023). Advances in design of polymer brush functionalized inorganic nanomaterials and their applications in biomedical arena. WIREs Nanomedicine and Nanobiotechnology.

[93] Wang R., Wei Q., Sheng W., Yu B., Zhou F., Li B. (2023). Driving polymer brushes from synthesis to functioning. Angew. Chem. Int. Ed..

[94] Khodadadi Yazdi M., Zarrintaj P., Saeb M.R., Mozafari M., Bencherif S.A. (2024). Progress in ATRP-derived materials for biomedical applications. Prog. Mater. Sci..

[95] Szewczyk-Łagodzińska M., Plichta A., Dębowski M., Kowalczyk S., Iuliano A., Florjańczyk Z. (2023). Recent advances in the application of ATRP in the synthesis of drug delivery systems. Polymers.

[96] Kiełbasa A., Kowalczyk K., Chajec-Gierczak K., Bała J., Zapotoczny S. (2024). Applications of surface-grafted polymer brushes with various architectures. Polym. Adv. Technol..

[97] Masuda T. (2024). Design of functional soft interfaces with precise control of the polymer architecture. Polym. J..

[98] Adeli F., Abbasi F., Ghandforoushan P., Külahlı H.E., Meran M., Abedi F., Ghamkhari A., Afif S. (2023). Recent advances in formulation and application of molecular polymer brushes in biomedicine: therapeutic, diagnostic, and theranostics capabilities. Nano Today.

[99] Matyjaszewski K., Xia J. (2001). Atom transfer radical polymerization. Chem. Rev..

[100] Ejaz M., Yamamoto S., Ohno K., Tsujii Y., Fukuda T. (1998). Controlled graft polymerization of methyl methacrylate on silicon substrate by the combined use of the Langmuir-blodgett and atom transfer radical polymerization techniques. Macromolecules.

[101] Kobayashi M., Terayama Y., Yamaguchi H., Terada M., Murakami D., Ishihara K., Takahara A. (2012). Wettability and antifouling behavior on the surfaces of superhydrophilic polymer brushes. Langmuir.

[102] Huang Y., Morinaga T., Tai Y., Tsujii Y., Ohno K. (2014). Immobilization of semisoft colloidal crystals formed by polymer-brush-afforded hybrid particles. Langmuir.

[103] Higaki Y., Kobayashi M., Takahara A. (2020). Hydration state variation of polyzwitterion brushes through interplay with ions. Langmuir.

[104] Yoshikawa C., Sakakibara K., Nonsuwan P., Yamazaki T., Tsujii Y. (2021). Nonbiofouling coatings using bottlebrushes with concentrated polymer brush architecture. Biomacromolecules.

[105] Inoue Y., Kim Y., Hasegawa H., Yoshida Y., Sakakibara K., Tsujii Y. (2023). A novel electrochemical biosensing method with double-layered polymer brush modified electrode. Colloids Surf., B.

[106] Inoue Y., Ishihara K. (2010). Reduction of protein adsorption on well-characterized polymer brush layers with varying chemical structures. Colloids Surf., B.

[107] Balamurugan S., Mendez S., Balamurugan S.S., O'Brien II M.J., López G.P. (2003). Thermal response of poly(*N*-isopropylacrylamide) brushes probed by surface plasmon resonance. Langmuir.

[108] Wu T., Zhang Y., Wang X., Liu S. (2008). Fabrication of hybrid silica nanoparticles densely grafted with thermoresponsive poly(N-isopropylacrylamide) brushes of controlled thickness via surface-initiated atom transfer radical polymerization. Chem. Mater..

[109] Turan E., Demirci S., Caykara T. (2010). Synthesis of thermoresponsive poly(N-isopropylacrylamide) brush on silicon wafer surface via atom transfer radical polymerization. Thin Solid Films.

[110] Yu Q., Zhang Y., Chen H., Wu Z., Huang H., Cheng C. (2010). Protein adsorption on poly(N-isopropylacrylamide)-modified silicon surfaces: effects of grafted layer thickness and protein size. Colloids Surf., B.

[111] Conzatti G., Cavalie S., Combes C., Torrisani J., Carrere N., Tourrette A. (2017). PNIPAM grafted surfaces through ATRP and RAFT polymerization: chemistry and bioadhesion. Colloids Surf., B.

[112] Nagase K., Kobayashi J., Kikuchi A., Akiyama Y., Kanazawa H., Okano T. (2015). Thermoresponsive hydrophobic copolymer brushes modified porous monolithic silica for high-resolution bioseparation. RSC Adv..

[113] Nagase K., Kobayashi J., Kikuchi A., Akiyama Y., Kanazawa H., Okano T. (2014). Monolithic silica rods grafted with thermoresponsive anionic polymer brushes for high-speed separation of basic biomolecules and peptides. Biomacromolecules.

[114] Nagase K., Yuk S.F., Kobayashi J., Kikuchi A., Akiyama Y., Kanazawa H., Okano T. (2011). Thermo-responsive protein adsorbing materials for purifying pharmaceutical protein on exposed charging surface. J. Mater. Chem..

[115] Gritti F., Trebel N., Höltzel A., Tallarek U. (2022). Prediction of surface excess adsorption and retention factors in reversed-phase liquid chromatography from molecular dynamics simulations. J. Chromatogr. A.

[116] Xiao D., Wirth M.J. (2002). Kinetics of surface-initiated atom transfer radical polymerization of acrylamide on silica. Macromolecules.

[117] Nagase K., Kobayashi J., Kikuchi A., Akiyama Y., Annaka M., Kanazawa H., Okano T. (2008). Influence of graft interface polarity on hydration/dehydration of grafted thermoresponsive polymer brushes and steroid separation using all-aqueous chromatography. Langmuir.

[118] Nagase K., Kobayashi J., Kikuchi A., Akiyama Y., Kanazawa H., Okano T. (2008). Effects of graft densities and chain lengths on separation of bioactive compounds by nanolayered thermoresponsive polymer brush surfaces. Langmuir.

[119] Feil H., Bae Y.H., Feijen J., Kim S.W. (1993). Effect of comonomer hydrophilicity and ionization on the lower critical solution temperature of *N*-isopropylacrylamide copolymers. Macromolecules.

[120] Yakushiji T., Sakai K., Kikuchi A., Aoyagi T., Sakurai Y., Okano T. (1998). Graft architectural effects on thermoresponsive wettability changes of poly(N-isopropylacrylamide)-Modified surfaces. Langmuir.

[121] Nagase K., Geven M., Kimura S., Kobayashi J., Kikuchi A., Akiyama Y., Grijpma D.W., Kanazawa H., Okano T. (2014). Thermoresponsive copolymer brushes possessing quaternary amine groups for strong anion-exchange chromatographic matrices. Biomacromolecules.

[122] Kikuchi A., Kobayashi J., Okano T., Iwasa T., Sakai K. (2007). Temperature-modulated interaction changes with adenosine nucleotides on intelligent cationic, thermoresponsive surfaces. J. Bioact. Compat Polym..

[123] Nagase K., Kobayashi J., Kikuchi A., Akiyama Y., Kanazawa H., Okano T. (2007). Interfacial property modulation of thermoresponsive polymer brush surfaces and their interaction with biomolecules. Langmuir.

[124] Mizutani A., Nagase K., Kikuchi A., Kanazawa H., Akiyama Y., Kobayashi J., Annaka M., Okano T. (2010). Preparation of thermo-responsive polymer brushes on hydrophilic polymeric beads by surface-initiated atom transfer radical polymerization for a highly resolutive separation of peptides. J. Chromatogr. A.

[125] Nagase K., Kitazawa S., Yamada S., Akimoto A.M., Kanazawa H. (2020). Mixed polymer brush as a functional ligand of silica beads for temperature-modulated hydrophobic and electrostatic interactions. Anal. Chim. Acta.

[126] Nagase K., Kobayashi J., Kikuchi A., Akiyama Y., Kanazawa H., Okano T. (2011). Thermally-modulated on/off-adsorption materials for pharmaceutical protein purification. Biomaterials.

[127] Nagase K., Mizutani Akimoto A., Kobayashi J., Kikuchi A., Akiyama Y., Kanazawa H., Okano T. (2011). Effect of reaction solvent on the preparation of thermo-responsive stationary phase through a surface initiated atom transfer radical polymerization. J. Chromatogr. A.

[128] Nagase K., Umemoto Y., Kanazawa H. (2021). Effect of pore diameter on the elution behavior of analytes from thermoresponsive polymer grafted beads packed columns. Sci. Rep..

