# Supplementary material for: Temperature-modulated interactions between thermoresponsive strong cationic copolymer-brush-grafted silica beads and biomolecules
Source: Heliyon. 2024 Jul 20;10(15):e34668. doi: 10.1016/j.heliyon.2024.e34668 (PMC11332852; doi:10.1016/j.heliyon.2024.e34668)
Supplement: Multimedia component 1 [file mmc1.docx]

*Supplementary Material*

Temperature-modulated interactions between thermoresponsive strong cationic copolymer brush grafted silica beads and biomolecules

Kenichi Nagase ^1，2^*, Sayaka Suzuki^2^, Hideko Kanazawa^2^

Author Affiliation

1. Graduate School of Biomedical and Health Sciences, Hiroshima University, 1-2-3 Kasumi, Minami-ku, Hiroshima, 734-8553, Japan
2. Faculty of Pharmacy, Keio University, 1-5-30 Shibakoen, Minato, Tokyo, 105-8512, Japan

*Corresponding author: Kenichi Nagase, PhD

E-mail: nagase@hiroshima-u.ac.jp, nagase.kenichi@keio.jp

Tel: +81-82-257-5323

**S.1 Materials**

*N*-isopropylacrylamide (NIPAAm) and 3-acrylamidopropyl trimethylammonium chloride (APTAC) were obtained from KJ Chemicals (Tokyo, Japan). The polymerization inhibitor of NIPAAm was removed via recrystallization using *n*-hexane. The polymerization inhibitor of APTAC was removed using an inhibitor–remover column (Sigma Aldrich, St Louis, MO, USA). *N*-*tert*-butylacrylamide (tBAAm) and *n*-butyl methacrylate (nBMA) were obtained from Fujifilm Wako Pure Chemical (Osaka, Japan). The polymerization inhibitor of tBAAm was removed via recrystallization using acetone. Tris(2-aminoethyl)amine (TREN), copper (I) chloride, copper (II) chloride, acetone, hydrochloride, 2-propanol, adenosine nucleotides, and phosphate-buffered powder (1/15 mol/L, pH: 7.0) were obtained from Fujifilm Wako Pure Chemical (Osaka, Japan). Tris[2-(dimethylamino)ethyl]amine (Me_6_TREN) was synthesized from TREN. ([Chloromethyl]phenylethyl) trimethoxysilane (CPTMS) and 3-aminopropyltrimethoxysilane (APTMS) were obtained from Gelest (Morrisville, PA, USA). Silica beads (average diameter: 5 μm; pore size: 30 nm; and specific surface area: 100 m^2^/g) were purchased from Macherey–Nagel (Düren, Germany). Insulin, insulin chain A, insulin chain B, γ-globulin, and human serum albumin were obtained from Sigma Aldrich (St Louis, MO, USA). Stainless steel columns (inner diameter: 4.6 mm and column length: 50 mm) were obtained from GL Science (Tokyo, Japan).

**S.2 Property of the analytes**

**Table S1.** Properties of the adenosine nucleotides

| Compounds | Structure | Molecular weight | LogP | p*K*_a_ |
| --- | --- | --- | --- | --- |
| AMP | 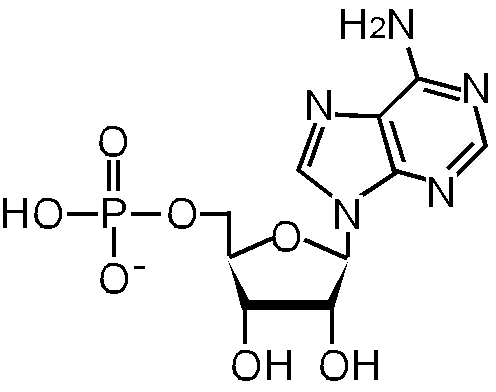 | 347.06 | −3.45 | 3.8 |
| ADP | 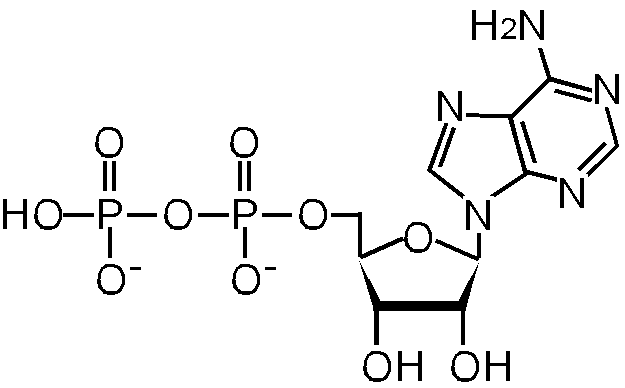 | 427.20 | −4.00 | 3.9 |
| ATP | 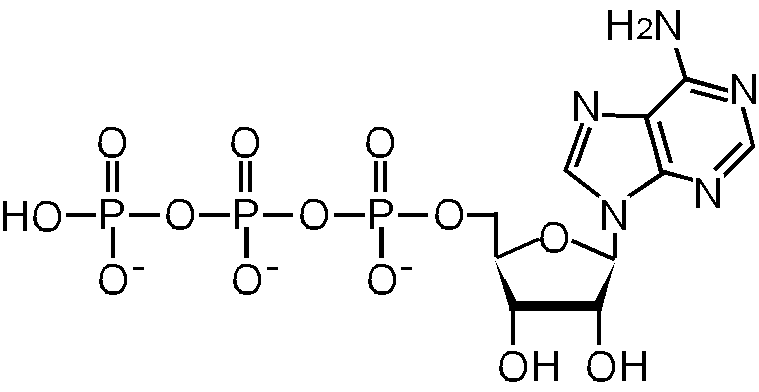 | 507.18 | −4.66 | 4.1 |

**Table S2.** Properties of insulin and its fragments

| Peptide | Amino-acid sequence | Molecular weight | Hydrophobicity (Σ*f*) | pI |
| --- | --- | --- | --- | --- |
| Insulin | GIVEQCCTSICSLYQLENYCN  FVNQHLCGSHLVEALYLVCGERGFFYTPKT | 5807 | 32.8 | 5.3 |
| Insulin chain A | GIVEQCCTSICSLYQLENYCN | 2530 | 11.04 |  |
| Insulin chain B | FVNQHLCGSHLVEALYLVCGERGFFYTPKT | 3496 | 24.05 |  |

**Table S3.** Properties of the proteins

| Protein | Molecular weight (kDa) | pI | Function |
| --- | --- | --- | --- |
| Albumin | 66.4 | 5.2 | Regulation of the colloidal pressure of blood |
| γ-Globulin | 155–160 | 6.85 | Specific interaction with antigen |


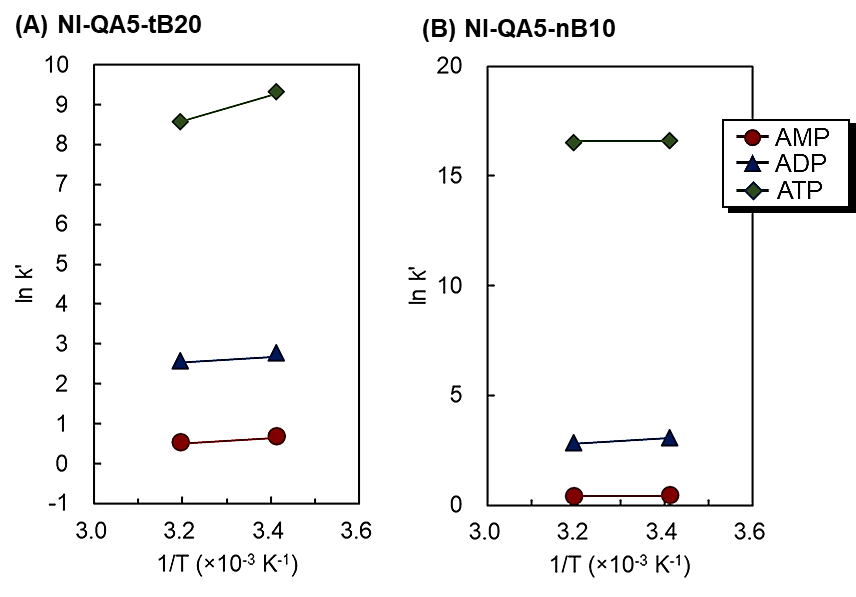


**Fig. S1** van’t Hoff plots of the adenosine nucleotides on the prepared columns: (A) P(NIPAAm-*co*-APTAC-*co*-tBAAm) brush-grafted silica bead-packed column (NI-QA5-tB20) and (B) P(NIPAAm-*co*-APTAC-*co*-nBMA) brush-grafted silica bead-packed column (NI-QA5-nB10)


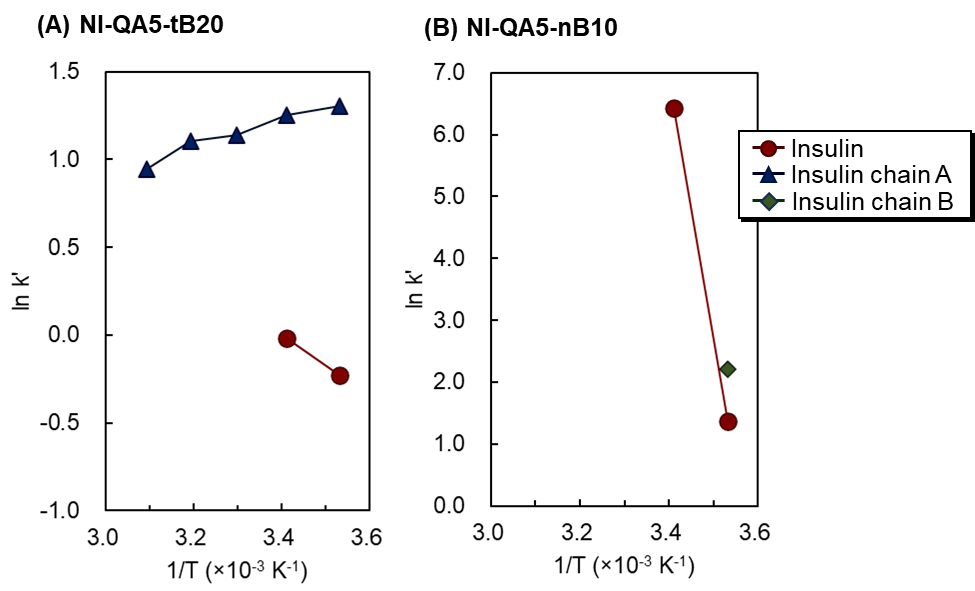


**Fig. S2** van’t Hoff plots of the insulin on the prepared columns: (A) P(NIPAAm-*co*-APTAC-*co*-tBAAm) brush-grafted silica bead-packed column (NI-QA5-tB20) and (B) P(NIPAAm-*co*-APTAC-*co*-nBMA) brush-grafted silica bead-packed column (NI-QA5-nB10)


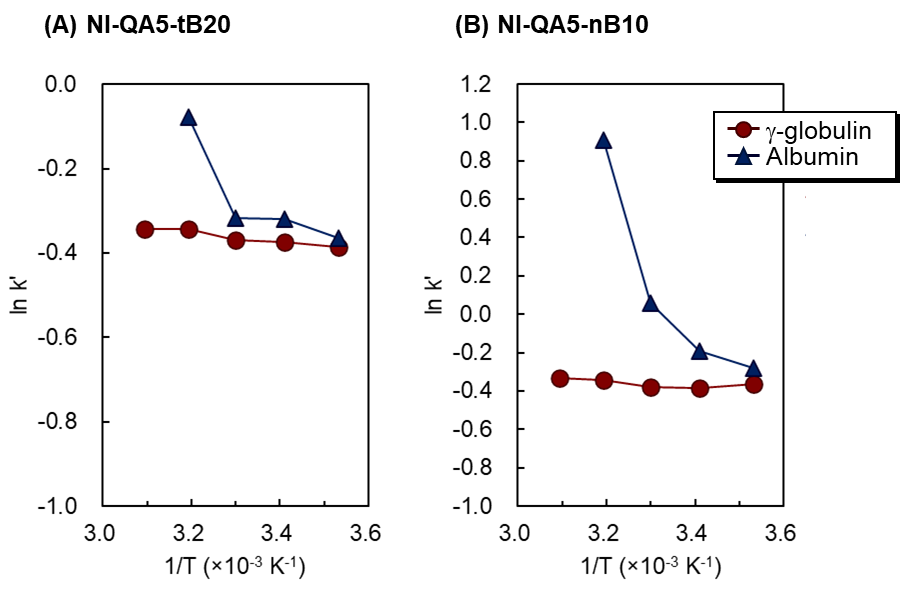


**Fig. S3** van’t Hoff plots of the protein on the prepared columns: (A) P(NIPAAm-co-APTAC-co-tBAAm) brush-grafted silica bead-packed column (NI-QA5-tB20) and (B) P(NIPAAm-co-APTAC-co-nBMA) brush-grafted silica bead-packed column (NI-QA5-nB10)
